# Transcriptional Reprogramming and Novel Therapeutic Approaches for Targeting Prostate Cancer Stem Cells

**DOI:** 10.3389/fonc.2019.00385

**Published:** 2019-05-09

**Authors:** Gianluca Civenni, Domenico Albino, Dheeraj Shinde, Ramiro Vázquez, Jessica Merulla, Aleksandra Kokanovic, Sarah N. Mapelli, Giuseppina M. Carbone, Carlo V. Catapano

**Affiliations:** Institute of Oncology (IOR), Università della Svizzera Italiana, Bellinzona, Switzerland

**Keywords:** prostate cancer, cancer stem cells, transcription factors, ERG, ESE3/EHF, c-Myc, STAT3, NF-κB

## Abstract

Prostate cancer is the most common malignancy in men and the second cause of cancer-related deaths in western countries. Despite the progress in the treatment of localized prostate cancer, there is still lack of effective therapies for the advanced forms of the disease. Most patients with advanced prostate cancer become resistant to androgen deprivation therapy (ADT), which remains the main therapeutic option in this setting, and progress to lethal metastatic castration-resistant prostate cancer (mCRPC). Current therapies for prostate cancer preferentially target proliferating, partially differentiated, and AR-dependent cancer cells that constitute the bulk of the tumor mass. However, the subpopulation of tumor-initiating or tumor-propagating stem-like cancer cells is virtually resistant to the standard treatments causing tumor relapse at the primary or metastatic sites. Understanding the pathways controlling the establishment, expansion and maintenance of the cancer stem cell (CSC) subpopulation is an important step toward the development of more effective treatment for prostate cancer, which might enable ablation or exhaustion of CSCs and prevent treatment resistance and disease recurrence. In this review, we focus on the impact of transcriptional regulators on phenotypic reprogramming of prostate CSCs and provide examples supporting the possibility of inhibiting maintenance and expansion of the CSC pool in human prostate cancer along with the currently available methodological approaches. Transcription factors are key elements for instructing specific transcriptional programs and inducing CSC-associated phenotypic changes implicated in disease progression and treatment resistance. Recent studies have shown that interfering with these processes causes exhaustion of CSCs with loss of self-renewal and tumorigenic capability in prostate cancer models. Targeting key transcriptional regulators in prostate CSCs is a valid therapeutic strategy waiting to be tested in clinical trials.

## Introduction

**Figure 1:**
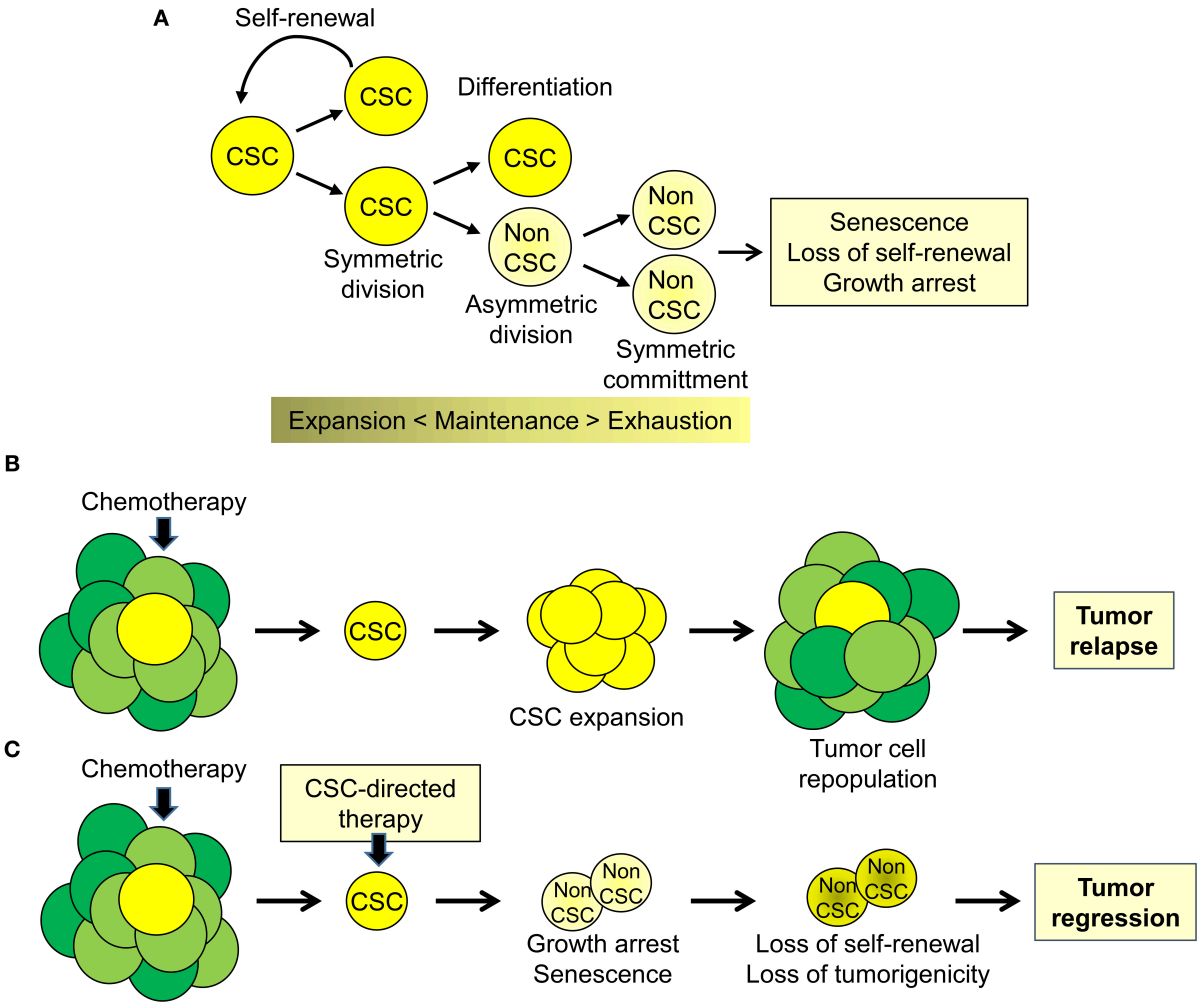
Cancer stem cell biology and perspectives for cancer therapy. **(A)** Cancer stem cell (CSC) are a subpopulation of tumor cells capable of self-renewing through symmetric cell division and of generating, through asymmetric division, more differentiated proliferating daughter cells (non-CSC) that, through successive cell divisions (symmetric commitment) constitute the bulk of the tumor mass. **(B)** CSC are intrinsically resistant to chemotherapy and other therapeutic modalities and cause disease recurrence by reconstituting the original tumor cell population at the primary or metastatic sites. **(C)** Targeting CSC could impair tumor regrowth and decrease the likelihood of tumor progression and disease recurrence.

To date there is compelling evidence supporting the presence of tumor-initiating, tumor-propagating stem-like cells or cancer stem cells (CSCs) in human cancers (1, 2). At any given time, the CSCs likely constitute only a minority of tumor cells within the tumor mass (1, 2). However, CSCs contribute substantially to the biological and clinical heterogeneity of human cancers (3, 4). The CSC model proposes that tumor cells maintain a lineage hierarchy similar to normal tissues (2). The small population of stem-like cancer cells that sustain this hierarchical organization is able both to self-renew by symmetric cell division and to produce, through asymmetric cell divisions, phenotypically distinct daughter cells with limited self-renewal but greater proliferative activity (Figure 1A). Similar to normal stem cells, the balance between self-renewal, differentiation, and senescence is essential to maintain the CSC subpopulation (2). Importantly, these processes lead to the expansion and maintenance or, alternatively, to progressive loss of proliferative potential and exhaustion of the CSC pool. According to the stem cell model, CSCs are key elements driving tumor heterogeneity and contributing to tumor progression and metastases (2, 4). Importantly, CSCs contribute substantially to treatment failure and disease recurrence by virtue of their intrinsic resistance to chemotherapy, radiotherapy, and even molecular-targeted drugs (2, 3, 5). Despite even massive reduction of bulk tumor cells after effective treatment, the CSC subpopulation can survive, expand and reconstitute, through a combination of symmetric and asymmetric cell divisions, the population of bulk tumor cells leading to tumor re-growth and relapse (Figure 1B). Indeed, the inability of current therapies to affect the CSC subpopulation contributes to their limited success and the almost inevitable progression to treatment-resistant disease. In this scenario, a significant increase in treatment efficacy, duration of clinical response, and patient survival may depend on the clinical implementation of new treatment strategies aimed at eliminating or reprogramming CSCs toward differentiation and senescence (Figure 1C). In this context, the knowledge of the pathways underlying the peculiar properties of CSCs can provide ideal targets for development of CSC-directed therapies (4–6).

In this review, we focus on prostate cancer and the role of transcriptional regulators on phenotypic reprogramming of prostate CSCs. We provide examples supporting the possibility of interfering with maintenance and expansion of the CSC subpopulation in human prostate cancer by targeting transcriptional regulators. Transcriptional and epigenetic regulatory factors are key elements for instructing specific transcriptional programs and phenotypic changes in CSCs (3, 6). Notably, recent work has established that interfering with these processes can induce loss of self-renewal capability and exhaustion of the tumorigenic potential of CSCs. Promising compounds are emerging from preclinical studies. Thus, targeting transcriptional regulators in prostate CSCs might be a valid therapeutic strategy to explore further in the preclinical and clinical setting.

## Prostate Cancer and the Current Treatment Perspective

Prostate cancer is the most common malignancy in men and the second cause of cancer-related deaths in developed countries (7). Despite the progress in the treatment of localized prostate cancer, management of locally advanced and metastatic disease is still a critical unmet need (8, 9). Recent genomic studies have shown that multiple genetic and epigenetic events contribute to prostate cancer initiation and progression (10–12). Deregulated expression and activity of transcriptional and epigenetic regulators occur at early stages of disease and are particularly relevant during the progression from localized to metastatic disease and development of treatment-resistant prostate cancer (13, 14). Moreover, complex transcriptional and epigenetic reprogramming contribute to cancer cell plasticity or trans-differentiation leading to the acquisition of tumorigenic, stem-like, mesenchymal, or neuroendocrine features (15–17).

The prostate is an exocrine gland that is located around the urethra at the base of the bladder and produces the alkaline seminal fluid (18). Histologically, the human prostate is composed of a pseudostratified epithelium containing basal and luminal epithelial cells with rare neuroendocrine cells (19, 20). Luminal cells are differentiated secretory epithelial cells that line the lumen of the ducts and secretes the alkaline prostatic fluid (20). Luminal secretory cells express cytokeratin 8, cytokeratin 18 and the androgen receptor (AR). Basal cells lie on the basement membrane between luminal cells. Basal cells have low levels of AR and express cytokeratin 5, cytokeratin 14 and p63. Basal cells are considered the main niche for stem and progenitor cells within the normal prostate epithelium, although more recent lineage-tracing studies suggest that both basal and luminal cells contain lineage-restricted stem/progenitor cells in the mouse prostate (19, 20). Rare neuroendocrine cells, which express chromogranin A and synaptophysin, are scattered in the prostate gland. Neuroendocrine cells are AR negative and androgen-independent (19).

Most prostate tumors are adenocarcinomas arising from the peripheral zone of the prostate gland (18). The majority of human prostatic adenocarcinomas have a predominant luminal phenotype, with a limited number of primary tumors showing features of neuroendocrine, small cell or sarcomatoid carcinomas. Some 15% of patients diagnosed with a prostate cancer will ultimately develop metastatic lesions, with about 90% of these cases presenting with osteoblastic bone metastases (18). In about 85% of the metastatic patients, the bone is the sole site of metastasis. Notably, aggressive prostate adenocarcinomas with neuroendocrine features (NEPC) form preferentially osteoclastic bone metastases and metastasize more frequently to brain, liver, bladder, and adrenal gland than adenocarcinoma-type tumors (16).

**Figure 2:**
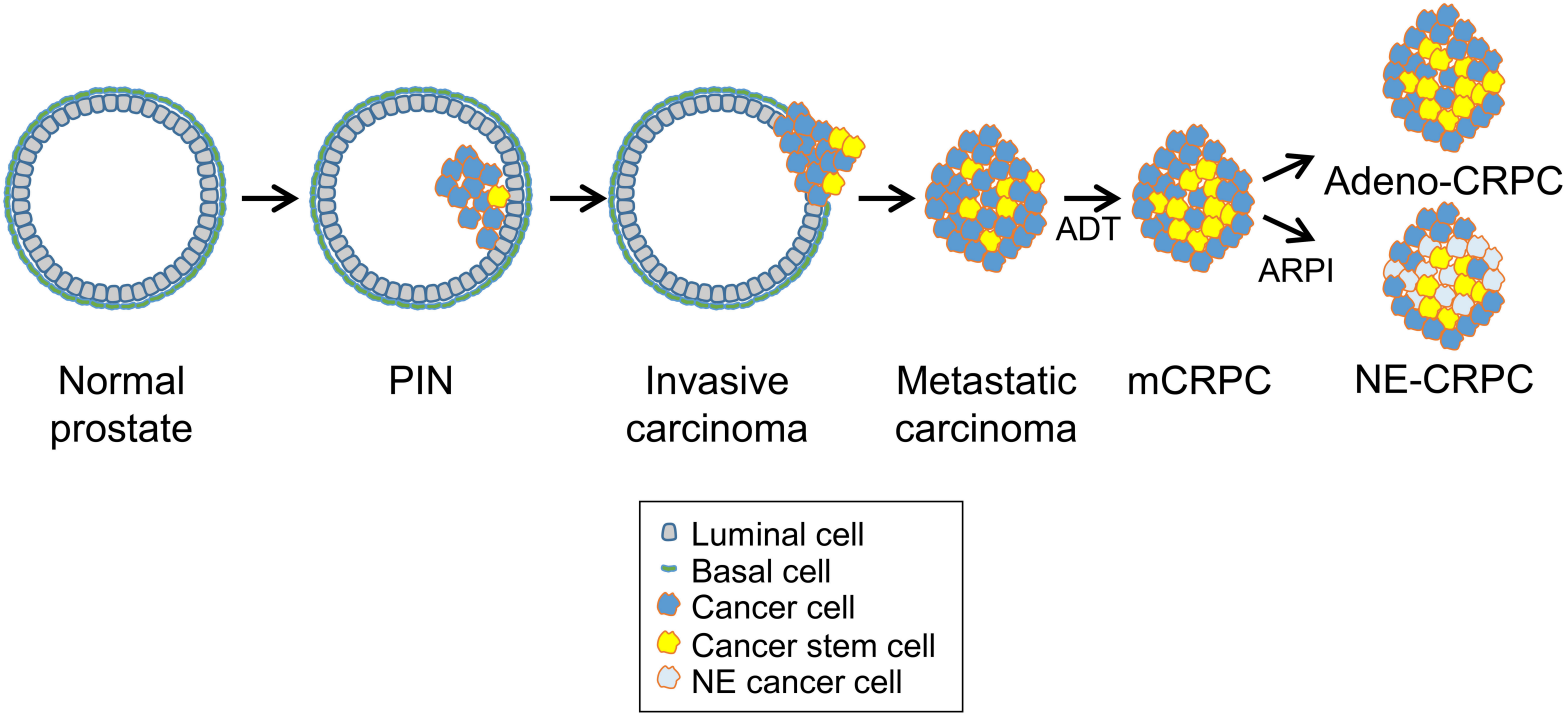
Prostate cancer progression and cancer stem cells. Prostate cancers initiate as *in situ* carcinoma called prostatic intraepithelial neoplasia (PIN) and then evolve into invasive carcinomas and later, after androgen deprivation therapy (ADT), progress to metastatic castration-resistant prostate carcinomas (mCRPC). After continuous ADT or treatment with new AR-pathway inhibitors (ARPI), treatment-resistant tumors emerge that either retain adenocarcinoma features with enhanced AR signaling (Adeno-CRPC) or acquire neuroendocrine features with attenuated AR signaling (NE-CRPC). Progression through these stages and development of castration-resistance are driven likely by the expansion and specific behavior of prostate cancer stem cells.

The clinical evolution of prostate cancer is highly heterogeneous, ranging from indolent to very aggressive tumors that rapidly progress to metastatic and treatment refractory prostate cancer (9, 18). Surgery and radiotherapy are highly effective for treatment of low-risk localized prostate tumors (21). Because most prostate cancers are androgen-dependent at the time of diagnosis, patients with locally advanced or metastatic diseases are treated with androgen deprivation therapy (ADT), which limits disease progression (Figure 2) (8). Nevertheless, most tumors eventually become resistant to ADT and progress to lethal metastatic castration-resistant prostate cancer (mCRPC), for which there are limited treatment options (22, 23). Despite the reduced efficacy of ADT, many mCRPC continues to have active AR signaling through a variety of mechanisms including AR gene amplification, splice variants, point mutations, transcriptional upregulation, ligand-independent activation, and increased androgen and dihydrotestosterone (DHT) synthesis by the adrenal glands or the tumor (9, 24). The continued reliance on AR signaling makes a fraction of mCRPC still potentially responsive to new AR pathway inhibitors (ARPI), such as the anti-androgen receptor antagonist enzalutamide and the androgen-biosynthesis inhibitor abiraterone (Figure 2) (8). However, mCRPC can activate additional escape mechanisms and become resistant to the AR-targeted drugs (9).

An emerging modality of escape from ADT is phenotypic plasticity with the acquisition of neuroendocrine features and expression of characteristic markers such as synaptophysin and chromogranin (15, 25, 26). This process involves a complex interplay of multiple signaling pathways linked to transcriptional activators (e.g., STAT3, MYC family members, SOX2) and epigenetic effectors (e.g., EZH2) (16). In this context, expansion of AR-indifferent CSCs followed by differentiation toward a NE phenotype leads to a progeny of poorly differentiated tumor cells insensitive to androgen ablation or suppression (Figure 2). Thus, chronic ADT can induce dedifferentiation or transdifferentiation in mCRPCs with the NEPC variant considerably increasing among patients with metastatic castration-resistant disease. Neuroendocrine differentiation may represent an extreme form of evolution of prostate adenocarcinomas to an androgen-independent status.

mCRPCs non-responsive to ADT and AR-targeted therapeutics are treated with chemotherapy (27). Docetaxel is now the standard therapy for these patients, although the beneficial effect in this setting is rarely durable (28). Many patients do not respond or, after an initial response, become refractory to the treatment. Patients with docetaxel-refractory tumors generally receive cabazitaxel, a second-generation taxane, or platinum (Pt)-based compounds such as cisplatin and carboplatin (21, 29). Chemotherapy with carboplatin, docetaxel, or cabazitaxel is currently the preferred treatment for patients presenting with low PSA/tumor burden ratio and rapid metastatic progression or features of small cell carcinoma or NEPC (28). Inevitably, rapid development of resistance severely limits the duration of response and efficacy of any form of treatment in these patients.

## Cancer Stem Cells in Prostate Cancer

Prostate cancer is highly heterogeneous in cell composition (19). The presence of stem-like tumor cells with tumor-propagating and metastasis-generating properties can greatly influence the biological heterogeneity, clinical progression and treatment response (19). CSCs within primary tumors are likely the main cause of metastatic spread and disease recurrence in prostate cancer patients (Figure 2). Moreover, expansion of CSCs, which are independent of AR signaling, can contribute to the development of castration-resistance as well as to reduced sensitivity to chemotherapy and radiotherapy (19, 20, 30, 31). Furthermore, CSCs that derive from basal or luminal-type progenitor/stem cells may exhibit different characteristics and contribute diversely to the biological and clinical heterogeneity of prostate tumors and their propensity to aggressive behavior and treatment resistance (19, 20, 31).

CSCs display three main characteristics: the ability to initiate tumor (tumorigenesis), to maintain their cellular properties in at least one daughter cell (self-renewal) and to reproduce the cellular composition of the original tumor (differentiation program) (32). Several studies provide evidence for the presence of self-renewing tumor-initiating stem-like cancer cells in prostate tumors (19). Putative CSCs can be purified using appropriate cell surface markers to define specific cell populations and their properties can be assessed using *in vitro* tumor-sphere and *in vivo* transplantation assays (33–36). Broad and heterogeneous sets of extracellular markers have been used to identify and isolate prostate CSCs (37, 38). However, the reproducibility and reliability in different settings and experimental models as well as the clinical relevance of most markers have not been demonstrated with any certainty (38). Increased expression of intracellular markers (e.g., ALDH), stem cell reprogramming factor, transcriptional and epigenetic regulators (e.g., Oct3/4, Sox2, Klf4, Nanog, Myc, BMI1) characterize prostate CSCs and provide additional tools for their identification (36, 37, 39–41).

**Figure 3:**
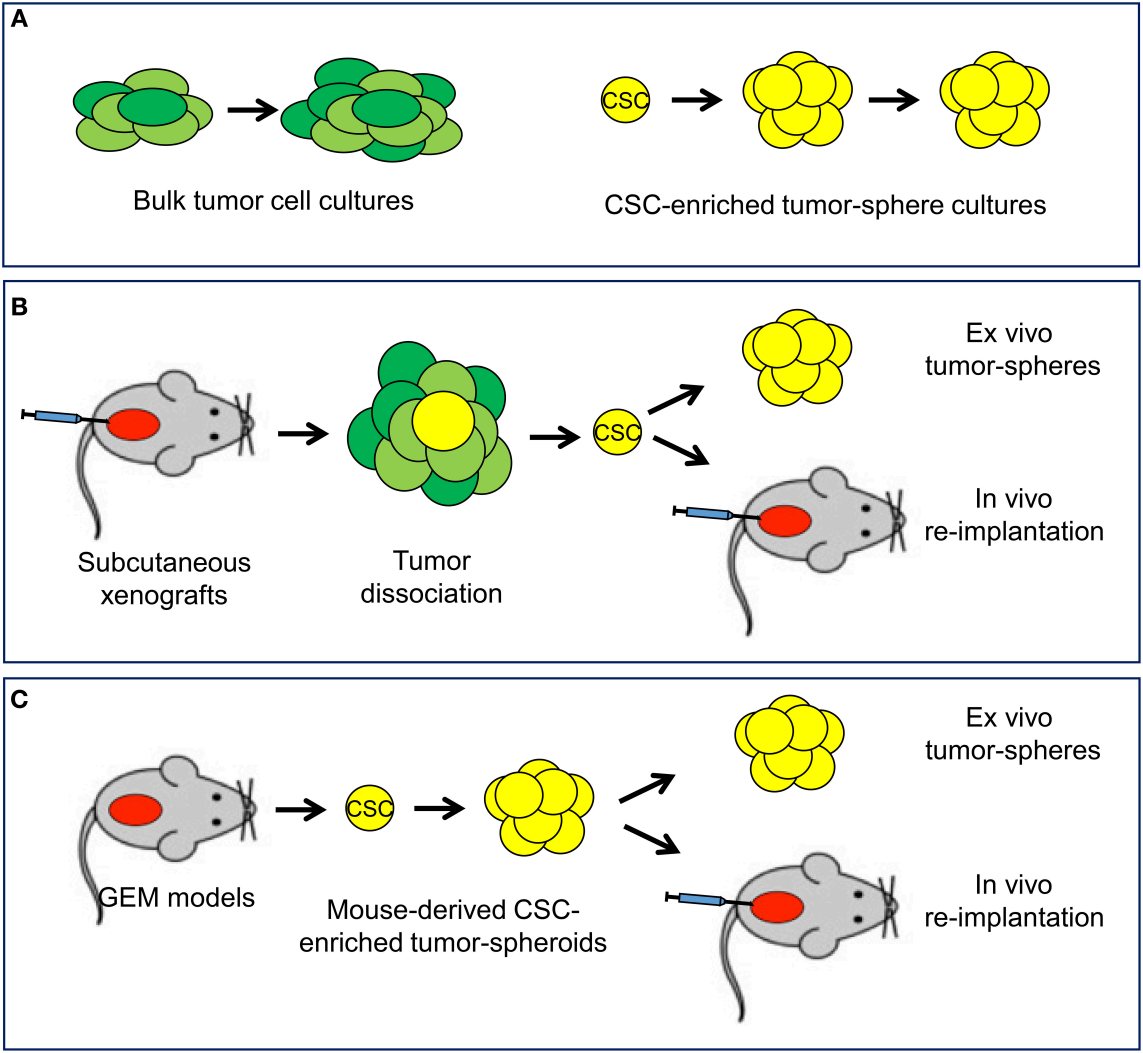
*In vitro* and *in vivo* experimental models available to study cancer stem cells. **(A)** *In vitro* systems include standard cultures of adherent bulk tumor cells and tumor-sphere cultures of cancer stem cell (CSC)-enriched subpopulation capable of self-renewal. **(B)** Mouse xenograft models allow the isolation of CSC and monitoring self-renewal and tumorigenicity in *ex vivo* tumor-sphere assays and *in vivo* serial re-implantation assays. **(C)** Tumor-sphere assays and *in vivo* serial re-implantation can be performed with genetically engineered mouse (GEM) models through the isolation and propagation of CSC-enriched tumor-spheroids.

In the experimental setting, *in vitro, ex vivo*, and *in vivo* functional assays are highly relevant to isolate CSCs and assess their content and properties (33–36, 42). Culturing prostate cancer cells in adherent monolayers in presence of serum-supplemented cell culture medium allows propagation of the heterogeneous bulk population of tumor cells (Figure 3A). Prostato-sphere or tumor-sphere cultures in serum-free liquid or semi-solid media and non-adherent conditions favors the expansion of single-cell derived colonies (spheroids), which are enriched of stem-like tumor cells able to survive and proliferate in this setting (34–36, 42). Organoid cultures derived from human or mouse tumors are an alternative method to preserve the heterogeneity of the cell composition of the original tumor and test drug efficacy in a three-dimensional, microenvironment-inclusive system (43, 44). However, unlike tumor-sphere culture systems, organoids do not enrich specifically for CSCs and do not allow a direct assessment of tumor cells with stem-like properties. Xenografts of established human cancer cell lines or patient-derived tumor cells by subcutaneous or orthotopic implantation in immunodeficient mice can be a reliable and reproducible source of stem-like tumor-initiating cells and are used to assess *in vivo* tumorigenicity and self-renewal properties of the isolated CSCs by serial re-implantation in mice (Figure 3B). Long-term tumor regeneration in mice as well as reproducible tumor-sphere forming ability *in vitro* are paramount evidence of stem-like capability of the isolated tumor cells (35, 36, 45). Furthermore, an emerging area of research involves the isolation, characterization and propagation of CSCs derived from genetically engineered mouse (GEM) models of prostate cancer (Figure 3C). These GEM models reproduce prostate tumors that mimic human cancer with similar defined genetic alterations within the orthotopic prostatic microenvironment and in presence of an intact immune system and thus are becoming a valuable resource to study prostate CSC behavior and response to treatment (19, 46, 47).

When properly applied, collectively, these experimental systems represent reliable tools to monitor the effects of genetic and pharmacological interventions on CSCs. Furthermore, these *in vitro/in vivo* assays along with supplementary approaches (e.g., gene signatures, surface markers) need to be implemented rigorously in preclinical and clinical studies to demonstrate the efficacy of CSC-directed strategies and monitor the dynamic changes in tumor cell subpopulations upon treatment (5, 48). Such studies would provide a great deal of essential information for defining the best strategies to improve cancer treatment in a precision medicine approach.

## Canonical Signaling Pathways in Prostate Cancer Stem Cells

Current therapies for prostate cancer target preferentially partially differentiated, AR-dependent and proliferating tumor cells that constitute the bulk of the tumor mass in locally advanced and metastatic tumors (8, 21, 28, 29). However, the subpopulation of CSCs is virtually insensitive to these therapies and can repopulate the tumors at primary and metastatic sites (19, 49). Understanding the pathways controlling the establishment, expansion and maintenance of the CSC pool would be an important step toward the development of more effective therapies for prostate cancer enabling the ablation or exhaustion of CSC and preventing treatment resistance and disease recurrence. Much emphasis has been put on canonical pathways identified as drivers of stemness features in normal stem cells and proven to have similar functions in CSCs.

Canonical stem cell-associated pathways, such as Sonic-Hedgehog, Wnt and Notch, play important roles in CSC maintenance and represent promising targets to explore for the eradication of prostate CSCs (50, 51). In the canonical Wnt pathway, Wnt ligands bind to Frizzled receptor and co-receptor LRP 5/6 leading to stabilization and nuclear translocation of β-catenin that acts as transcriptional activator of the expression of pro-tumorigenic genes (50). Altered expression and localization of β-catenin is frequent in advanced prostate cancer and the Wnt signaling pathway can directly promote self-renewal of prostate CSCs (52–55). The Hedgehog pathway controls cell renewal and survival in normal stem cells during embryogenesis and adulthood (50). Hedgehog signaling is activated by binding of a specific set of ligands (Desert, Indian and Sonic) to the membrane receptors Patched (Ptch1 and 2) and Smoothened (SMO). In the presence of the ligands, SMO is relieved from the repression by PTCH and promotes the nuclear translocation of transcription factor Gli, which triggers the expression of specific target genes. Prostate tumors, like other cancers, frequently exhibit abnormally activated Hedgehog signaling (56, 57), which promotes the expansion of prostate CSCs (58, 59). A complex set of receptors (Notch1-4) and ligands (DLL 1, DLL 3, DLL 4, Jagged 1, and Jagged 2) controls Notch signaling (50). Upon ligand binding, the cytoplasmic domain of the receptor is cleaved by proteolytic enzymes (ADAMs and γ-secretase) leading to the release of the Notch intracellular domain (NICD), which moves in the nucleus and activates transcription of target genes. The Notch signaling pathway is activated improperly in human cancers, including prostate tumors, where it alters normal differentiation programs and contribute to CSC expansion (50, 60–62). In prostate cancer, combined upregulation of Notch and Hedgehog signaling promotes the stem-like phenotype and treatment resistance (63, 64).

Inhibitors of the Hedgehog, Wnt and Notch pathway have been developed and some have been tested in clinical trials in oncological patients (50). Targeting these stemness-related pathways with selective inhibitors has potent anti-CSC effect and influences positively the response to other cancer treatments in preclinical models (65, 66). Notch pathway inhibitors have shown efficacy enhancing the activity of both chemotherapy and ADT in prostate cancer preclinical models (67–71). Hedgehog inhibitors have anti-CSC effects in prostate cancer reducing expression of stemness-related genes and growth of tumor xenografts in mice (63, 72–74). Wnt pathway inhibitors also have been tested successfully in preclinical models of prostate cancer, although the evidence of a direct anti-CSC effect is not systematically provided (54, 65, 66, 75). Wnt inhibitors include promising compounds that have shown relevant activity in various experimental cancer models (53, 76–78).

Ongoing trials in multiple cancer types, including prostate cancer, are testing the efficacy of canonical stemness pathway inhibitors (48, 79, 80). Notably, vismodegib (GDC-0449), the first inhibitor approved for clinical use, and other Hedgehog inhibitors are in clinical trials for prostate cancer patients. Similarly, several Wnt and Notch pathway inhibitors are currently undergoing clinical evaluation for treatment of patients with various types of tumors including patients with prostate cancer (80–82). These early phase clinical studies are set to determine the efficacy and toxicity of these compounds and they will provide insightful information for further development as single agents or in combinatorial regimens (48). However, it would be important in the future to assess specifically whether the compounds affect the prostate CSC subpopulation taking advantage of some of the assays developed in preclinical studies. It might also be difficult to exclude effects of these pharmacological pathway inhibitors on normal stem cells and prevent toxicity due to a limited therapeutic window (5, 48).

## Transcriptional Regulators in Prostate CSCs

Prostate CSCs present over-expression of various transcriptional and epigenetic regulators (e.g., Nanog, SOX2, BMI1, and EZH2) that are directly involved in reprogramming the CSC transcriptome and sustaining the stem-like phenotype. Some of these factors have been effectively targeted to induce CSC depletion and counteract treatment resistance (41, 83–87). Small molecule inhibitors of EZH2 and BMI1, two epigenetic effectors, are available and have shown efficacy in prostate cancer preclinical models (25, 26, 85, 88–92). Furthermore, EZH2 inhibitors are undergoing clinical testing in patients with advanced tumors.

Additional transcriptional regulators are emerging as targetable elements in prostate CSCs opening new opportunities for anti-CSC therapeutic interventions. In the following sections, we describe the recent data and provide proof of principle examples of the effectiveness of such approaches for targeting prostate CSCs.

### c-Myc

Additional pathways controlling the enhanced self-renewal capability and reduced differentiation potential of CSCs could provide ideal targets for development of CSC-specific treatment strategies. Several transcription factors aberrantly activated in advanced and mCRPCs can be directly responsible for expansion and tumorigenic potential of prostate CSCs. c-Myc (Myc) is a transcription factor involved in many biological processes, including transcription, replication, cell division, protein synthesis and metabolism (93). Amplification, chromosomal translocations, and deregulated expression of Myc are among the most common alterations occurring in human cancers (93). Myc is frequently upregulated in primary and metastatic prostate cancers and its overexpression has been associated with progression to CRPC (94). Many lines of evidence suggest that Myc has an important role in ensuring tumor development and maintenance of CSCs in human cancers (31, 95–97). Myc, along with other stem cell genes like SOX2, BMI1 and OCT-4, is highly expressed in prostate cancer cells having the CD44+/CD24– phenotype, which is considered a hallmark of stem/progenitor cells (36, 98). However, similar to many other transcription factors, Myc is a difficult target to address directly with conventional small-molecule drugs (99). Various approaches have been attempted to target Myc by blocking Myc-protein interactions, Myc-DNA interactions and Myc transcription or translation using small molecules, peptides, oligonucleotides and small interfering RNAs (99–102). Few compounds inhibiting Myc have entered early phases of clinical investigation (100).

Following previous studies on Myc transcription and promoter regulation by oligonucleotide-based approaches (103, 104), we showed more recently that Myc transcription could be epigenetically silenced using a novel strategy based on promoter-targeting siRNAs (105). This approach relies on the presence of a cis-acting non-coding promoter-associated RNA (paRNA) overlapping the gene transcription start site and positively regulating Myc gene transcription (105). siRNA directed to this paRNA inhibited Myc transcription by interfering with the formation of the transcription pre-initiation complex at the gene promoter (105). This strategy resulted in prolonged repression of Myc transcription. Interestingly, a single transfection of prostate cancer cells with the promoter-targeting siRNA induced long-lasting effects on cell proliferation and colony formation in CRPC models such as the DU145 and PC3 cell lines, indicative of persistent loss of proliferative potential as consequence of Myc silencing (105). Notably, using this promoter-targeting strategy we were able to show that Myc silencing impaired maintenance and induced senescence in the prostate CSC subpopulation blocking their expansion and tumorigenic potential (36). We showed that tumor-sphere forming cells derived from these human cancer cell lines and grown in stem cell selective conditions retained high self-renewal capability and had high tumorigenic potential and ability to reconstitute the original tumor cell population. Myc silencing impaired propagation of tumor-spheres *in vitro*, growth of subcutaneous tumors and formation of metastasis in mice (36). Consistent with an impact on CSCs, tumors formed by Myc-depleted cells had reduced content of stem-like tumor cells capable of forming *ex vivo* tumor-spheres and generating secondary tumor xenografts in mice. Thus, these *ex vivo* assays provided direct confirmation of the anti-CSC effect of Myc silencing. Notably, the reduced CSC content and tumorigenic capability was associated with increased senescence in CSCs both *in vitro* and *in vivo*. Thus, Myc silencing led to depletion of CSCs and reduced their tumorigenic and metastatic potential through the activation of a latent senescence program in CSCs (36). This study, thus, provided direct evidence of the role of Myc in the maintenance of CSCs in human tumors and identified loss of self-renewal and induction of senescence as primary mechanisms of the depletion of tumor-initiating and metastatic prostate CSCs. These data also demonstrated that RNAi-based targeting of regulatory non-coding RNA could be an effective strategy to modulate gene expression for therapeutic applications.

Targeting upstream regulators or downstream effectors of Myc is also a valid approach (100, 102). Notably, bromodomain and extra-terminal domain (BET) proteins, such as BRD4, bind to acetylated histones and cooperate with multiple oncogenic transcription factors including Myc (106). Importantly, chemical inhibitors designed to disrupt BET protein-chromatin interactions interfere with expression and activity of Myc and other transcription factors (107–110). BET inhibitors are effective anticancer agents in preclinical models of multiple types of cancers (111). Currently, several BET protein inhibitors (e.g., ZEN003694, OTX015/MK-8628, ABBV-075, INCB057643, GSK525762/I-BET762, GS-5829) are in phase I/II clinical trials, with some studies specifically assessing their efficacy in prostate cancer patients alone or in combination with AR-targeted therapies (112). In prostate cancer, BET protein inhibitors modulate AR signaling and enhance the anti-androgenic effect of AR-targeted therapies in AR positive prostate cancer cells such as VCaP and LNCaP cells, making them suitable drugs for treatment of mCRPCs (113–116). Interestingly, BET protein inhibitors interfere with Myc functions in preclinical cancer models (106, 108, 110) and, therefore, have the potential to inhibit Myc-dependent processes also in prostate CSCs.

### STAT3

Signal transducer and activator of transcription 3 (STAT3) is a key element in multiple signaling pathways and is activated aberrantly in many human cancers (117–120). Phosphorylation at tyrosine 705 (Tyr705), which is catalyzed by protein tyrosine kinases such as Janus kinases (JAK), regulates STAT3 transcriptional activity by inducing dimerization of STAT3 monomers, nuclear accumulation and DNA binding (117, 118). The IL-6/JAK pathway is the main responsible of Tyr705 phosphorylation and activation of this pathway contributes to tumor development in many experimental models (117, 118). STAT3 activation is associated with advanced disease, metastasis and clinical progression (118). Despite some recent controversial observations, the evidence of a role of this transcription factor in tumorigenesis in clinical and experimental systems and of its potential as therapeutic target is rather overwhelming [for an extensive discussion of these issues see (119–121)].

Increasing evidence indicates that STAT3 also localizes to mitochondria and is important in controlling mitochondria function (120, 122, 123). Mitochondrial STAT3 is phosphorylated at serine 727 (Ser727) by various serine protein kinases, whereas nuclear STAT3 is predominantly phosphorylated at Tyr705 by tyrosine protein kinases, like JAK family kinases (124, 125). Interestingly, constitutively Ser727-phosphorylated STAT3 is present in many human cancers and is sufficient to drive tumorigenesis independent of Tyr705 phosphorylation in various models (124, 126, 127). Moreover, mitochondrial STAT3 is critical for survival of tumor cells under microenvironment or treatment induced stress conditions, reflecting a tumor-specific dependency on STAT3 mitochondrial functions (124, 128).

A large set of evidence reveals a critical role of the STAT3 in prostate cancer. Over-activity of STAT3 in human cancers, including prostate cancer, is frequently the result of deregulation of upstream pathways with activation of protein tyrosine kinases associated with cytokine and growth factor receptors, like JAK family kinases (123). Increased levels of IL-6, IL-6 receptor, JAK1, and pSTAT3 have been detected in patients with metastatic tumors and CRPCs (129, 130) and are associated with poor prognosis (131, 132). The IL-6/JAK/STAT3 pathway contributes to treatment resistance promoting tumor cell survival after targeted anticancer drugs or ADT (133, 134). The pathway is at the center of tumor-microenvironment crosstalks that promote treatment resistance and stemness (134, 135). Activation of STAT3 can promote also immune-tolerance and chemo-resistance in prostate cancer through the secretion of immunosuppressive cytokines in the tumor microenvironment (136). Conversely, inhibition of the IL-6/JAK/STAT3 pathway reduces tumor cell proliferation and restores sensitivity to AR-targeted drugs (137–139). Importantly, in recent years antibodies (e.g., siltuximab) targeting the IL-6/JAK/STAT3 pathway have been tested as monotherapy or in combination with cytotoxic drugs in various clinical trials for treatment of cancers, including prostate cancer (119, 140–142). However, despite the positive data in preclinical models, the clinical activity in advanced prostate cancer patients was modest or not significant (119, 140–142), suggesting that anti-IL-6 therapies may not be the most effective approach to block STAT3 signaling in this setting.

Increased STAT3 levels and higher Tyr705 and Ser727 phosphorylation are frequent in human prostate cancer both at early (androgen-dependent) and late (castration-resistant) stages of the disease (143). STAT3 activation is associated with poor clinical outcome in prostate cancer patients (144, 145). Importantly, activation of STAT3 has been associated with promotion and maintenance of CSC, tumorigenicity and metastatic capability in prostate cancer (133, 146, 147). Alternative activation pathways and non-transcriptional functions of STAT3 may also be important in CSC maintenance (122). In prostate cancer, induction of Ser727 phosphorylation can promote cell transformation and tumor development in the absence of Tyr705 phosphorylation (126). The oncogenic effect of STAT3 in this experimental system depended strictly on phosphorylation of Ser727 and both transcriptional dependent and independent functions of STAT3 (126). Interestingly, we found that in a subset of prostate cancer, characterized by reduced expression of the ETS factor ESE3/EHF, STAT3 upregulation and activation depended on the over-expression of a microRNA, miR-424, which prevented proteasomal degradation of STAT3 and led to increased levels of total STAT3 protein (148). Remarkably, miR-424 upregulation correlated with the acquisition of CSC features in cell lines and human tumors, confirming the relevance of this non-canonical STAT3 activation pathway for stemness and tumorigenicity of prostate CSC (148).

The anti-CSC effects of interfering with IL-6/JAK signaling using chemical inhibitors or soluble IL-6R support the relevance of STAT3 activation in the CSC compartment (133, 146, 149–152). Napabucasin (BBI608), a small molecule inhibitor proposed to interfere with STAT3 signaling, has been shown to inhibit stem-like tumor cells in *ad-hoc* designed preclinical models (153). The compound has been extensively studied in preclinical setting as single agent and in drug combinations to take advantage of the concomitant targeting of CSC and non-CSC populations of tumor cells and is currently tested in several clinical trials in combination with standard therapies for advanced cancers (152, 154). In addition to STAT3 pathway or indirect inhibitors, various direct STAT3 inhibitors have been developed and some have been tested in prostate cancer models (119). We recently showed that small molecule inhibitors of STAT3 OPB-31121 and OPB-51602, which directly bind to the SH2 domain and effectively block global downstream signaling through multiple STAT3-dependent pathways, were very active in prostate cancer cell models and specifically highly effective on the CSC compartment (128, 155). OPB-31121, OPB-51602 and a third structurally related compound, OPB-111077, have entered phase I/II clinical trials showing some limited efficacy as single agents in advanced patients with solid tumors (156–159). These inhibitors block both Tyr705 and Ser727 phosphorylation and impair functioning of both nuclear and mitochondrial STAT3 (128). Importantly, in DU145 tumor xenografts, a CRPC model, OPB-51602 profoundly inhibited tumor growth and blocked tumor cell re-population after treatment withdrawal (128). These effects correlated with significant depletion of the fraction of stem-like tumor cells in the tumor xenografts after OPB-51602 treatment as assessed by *ex vivo* flow cytometry and tumor-sphere assays (128).

In human cancers, STAT3 activation occurs often concomitantly with activation of the NF-κB transcription factor pathway (160). NF-κB is frequently activated in advanced prostate cancer and has been implicated in expansion of CSC (37). Notably, STAT3 and NF-κB induce highly overlapping sets of pro-tumorigenic genes that might have important functions in prostate CSC (160). Activation of NF-κB and crosstalk with the IL6/JAK/STAT3 signaling pathway were essential for the acquisition of the epithelial-to-mesenchymal transition (EMT) and CSC features in aggressive prostate tumors (161). Furthermore, multiple positive and negative feedback loops link the two pathways leading to reciprocal activation or inhibition, depending on the cell context and microenvironment stimuli (160). Interestingly, we found that the activity of both STAT3 and NF-κB was strikingly higher in prostate CSC compared to bulk tumor cells and took advantage of the availability of a novel chimeric multi-kinase inhibitor, EC-70124, generated by genetic engineering of biosynthetic pathway of natural compounds (151). The novel compound was particularly effective against IKKβ and JAK kinases, which catalyze the critical steps for activation of NF-κB and STAT3, respectively (162). Thus, we reasoned that the ability of EC-70124 to target concomitantly NF-κB and STAT3 could provide an innovative strategy to disrupt the pro-tumorigenic crosstalk between the two transcription factors and avoid the downsides of individual pathway targeting and activation of alternative survival pathways. EC-70124 blocked effectively both NF-κB and STAT3 activity in prostate cancer cells and particularly in tumor-sphere cells with constitutive activation of these pathways (151). Moreover, the drug reduced tumor-sphere formation *in vitro* and tumor growth *in vivo* (151). Notably, EC-70124 had profound effect on the CSC subpopulation in tumor xenografts. This latter aspect was investigated by performing *ex vivo* assays with cells directly isolated from tumor xenografts at the end of the *in vivo* treatment and determining the fraction of tumor cells retaining CSC features and self-renewal capability (151). Thus, dual inhibition of STAT3 and NF-κB by EC-70124 impairs CSC maintenance and tumor development in mice and provides the basis for new therapeutic strategies for treatment of prostate cancer.

### ERG

ETS transcription factors constitute a large family of transcriptional regulators with important roles in cell differentiation and carcinogenesis (163). The ETS family includes 27 members that share the highly conserved ETS domain (163). Individual ETS factors have different patterns of cell and tissue specific expression and induce distinct transcriptional and biological responses. This diversity among individual ETS factors are reflected in different roles in tumorigenesis (163). ETS factors are deregulated in many human cancers and can either promote or suppress tumorigenesis (163).

A significant percentage of prostate cancers exhibit a specific gene fusion of the ETS gene ERG and the 5′ region of TMPRSS2 gene (164). The TMPRSS2 gene encodes a serine protease highly expressed in the prostate epithelium. This genetic event results in overexpression of full length (or minimally truncated) ERG protein driven by the androgen-regulated TMPRSS2 promoter in prostate epithelial cells (164–166). Interestingly, recent studies indicate the new options for targeting pharmacologically ERG for prostate cancer treatment (167–169). Ectopic expression of ERG results in complex changes in the cell transcriptome and acquisition of tumorigenic properties. However, the biological impact of aberrantly expressed ERG in prostate cancer progression and the underlying mechanisms are still unclear (170, 171). In a relevant number of human prostate cancers, ERG gene fusion occurs concomitantly with PTEN loss (172). The coexistence of the two events is generally associated with a more aggressive disease (172). Importantly, the cooperation of ERG gain and PTEN loss was recapitulated in mouse models whereby ERG transgenic mice crossed with PTEN-deficient mice developed frank malignant lesions and progression to invasive adenocarcinomas (172–174).

We recently used these GEM models with prostate-specific expression of ERG (Pb-Cre4; Rosa26ERG/ERG) with and without PTEN deletion to examine the mechanisms underlying tumor progression in ERG-fusion positive prostate cancers. ERG transgenic mice fail to develop invasive adenocarcinomas while the combined ERG/PTEN (Pb-Cre4; Ptenflox/flox; Rosa26ERG/ER) mice develop large invasive tumors (172). Thus, these GEM models represent good systems to assess events associated with prostate cancer progression. Moreover, to examine the relation between tumor progression and CSC, we took advantage of the established protocols for isolation and analysis of tumor-propagating stem-like tumor cells from *in vivo* models. Importantly, we found that prostate tumors from ERG/PTEN mice were highly enriched of stem-like cancer cells that formed large tumor-spheroids when plated in prostate-sphere culture conditions (47). Tumor-spheroids were positive for cytokeratins confirming their epithelial origin and expressed typical stem cell markers. Moreover, the ERG/PTEN derived tumor-spheroids were endowed of high *in vitro* self-renewal potential and were capable of generating tumors with high efficiency when re-implanted in mice (47).

Using this system, we recently evaluated the activity of compounds that could interfere with ERG induced transcriptonal and phenotypic reprogramming. Based on the finding of a relevant fraction of ERG/Sp1 co-regulated genes among the ERG activated targets in ERG-fusion positive tumor, we tested the activity of a novel DNA binding and Sp1 interfering compound, demycarosyl-3D-β-D-digitoxosyl-mithramycin SK (EC-8042), in ERG positive models (47). Specifically, we found that EC-8042 was a potent inhibitor of tumor-sphere formation by ERG fusion positive VCaP cells, a measure of the drug's anti-CSC activity. Interestingly, this effect was associated with reduced expression of ERG/Sp1 target genes and impaired invasive and metastatic property *in vivo* in the Chick Chorioallantoic Membrane (CAM) system (47). The CAM assay provide a simplified system to assess tumor growth, invasion, migration, circulation in blood vessels and metastasis in live chicken embryos. To investigate further the impact of EC-8042 on tumor-propagating stem-like cells, we took advantage of the ERG/PTEN GEM model. Treatment with EC-8042 reduced formation of tumor-spheroids from ERG/PTEN mice *in vitro* and impaired the re-implantation of tumor-spheroid cells in mice (47). Systemic treatment with EC-8042 inhibited tumor progression reducing invasive and proliferative areas in prostate adenocarcinomas in ERG/PTEN mice. Moreover, EC-8042 had a significant impact of the CSC subpopulation in ERG/PTEN mice as indicated by reduced *ex vivo* tumor-sphere formation and CSC marker expression (47). These data established for the first time the efficacy of antagonizing ERG oncogenic activity to block maintenance and expansion of CSC in ERG positive prostate tumor models opening new possibilities for treatment of this disease.

### ESE3/EHF

ESE3/EHF is an ETS family transcription factor of the epithelial-specific subfamily. ESE3/EHF is highly expressed in normal prostate epithelial cells and is essential for epithelial cell differentiation. Interestingly, we found that ESE3/EHF, along with ERG, was one of the most frequently deregulated ETS factors in human prostate cancer (175, 176). Importantly, down-regulation of ESE3/EHF in immortalized human prostate epithelial cells resulted in transformation, dedifferentiation, EMT, and acquisition of CSC properties (35). Furthermore, we identified a group of prostate tumors that exhibited marked reduction of ESE3/EHF expression in the absence of alterations of other ETS genes, including ERG. Enrichment of transcriptional features associated with EMT and CSC phenotype along with adverse clinical outcome characterized tumors with loss of ESE3/EHF expression (35). In follow up studies, we made further progress in understanding the tumor suppressor role of ESE3/EHF, particularly with respect to its function in cell differentiation and stemness. The link between ESE3/EHF and CSC properties was investigated by *in vitro* tumor-sphere and *in vivo* xenograft re-implantation assays (35, 45). ESE3/EHF knockdown in immortalized normal prostate epithelial cells, such as RWPE1 and LHS, was a potent inducer of stem-like, tumorigenic and self-renewal capability in prostate epithelial cells (35). Furthermore, we established that ESE3/EHF controls key genes and microRNAs specifically involved in epithelial differentiation and CSC maintenance (35, 45, 148). Collectively, these findings suggested also various strategies to target tumors with loss of ESE3/EHF expression and reverse their aggressive phenotype.

We found that ESE3/EHF downregulation led to increased expression of stem cell factors Lin28A/B along with other stemness-related factors (45). Lin28 A/B are key elements in the processing of mature microRNA of the let-7 family, which are potent tumor suppressors and anti-CSC effectors (177). Accordingly, we evaluated the effect of knocking down Lin28 on tumorigenic and stem-like properties of transformed prostate epithelial cells with ESE3/EHF downregulation. Lin28 knockdown reduced the expression of CSC markers and the ability to sustain tumor formation in mice (45). Accordingly, *ex vivo* tumor-sphere assays showed a significant and persistent reduction of stem-like cells in Lin28-depleted tumor xenografts. Moreover, in serial re-implantation experiments Lin28 knockdown decreased profoundly the *in vivo* self-renewal and tumorigenic potential of prostate CSC (45). Thus, targeting Lin28 could re-activate a latent differentiation/senescence program in prostate CSC and lead to their ablation in ESE3/EHF^low^ prostate tumors (45). Based on these findings, we recently evaluated a first chemical inhibitor of Lin28A/B, ID1632, and demonstrated *in vitro* its significant activity in CSC culture systems (178), suggesting an alternative to the siRNA and short-oligonucleotide based approaches (45, 179). All these modalities to target Lin28A/B and counteract the effects of ESE3/EHF silencing are in early preclinical stages of investigation.

We observed that ESE3/EHF had also a relevant impact on the activation state of STAT3. By performing miRNA expression profiling in a cohort of primary prostate tumors and normal prostate for which we had matching gene expression data we found that many microRNAs were significantly deregulated in tumors compared to normal prostate. We identified miR-424 as one of the most upregulated miRNAs in ESE3^low^ tumors and cell lines (148). Functional assays demonstrated that ESE3/EHF repressed transcription of miR-424 in normal prostate epithelial cells and loss of ESE3/EHF triggered miR-424 upregulation in cancer cells (148). Among the potential targets of miR-424, we found that the E3 ubiquitin ligase COP1 had a key role in miR-424 induced phenotypes in ESE3/EHF under-expressing prostate tumor cells. Interestingly, follow up studies revealed that miR-424 mediated silencing of COP1 led to impaired proteasomal degradation of STAT3 leading to stabilization and constitutive activation of this oncogenic transcription factor (148). Importantly, miR-424 upregulation promoted EMT and tumor-sphere formation, features associated with the CSC phenotype. Moreover, a synthetic antagonist of miR-424 reduced tumor-sphere formation *in vitro* and impaired the ability to generate tumors in mice (148). Several miRNA-based therapeutics are currently in clinical trials and represent promising tools for targeting oncogenic and tumor suppressor pathways (180).

ESE3/EHF modulates STAT3 activity also by controlling IL-6 transcription (150). We observed that expression of ESE3/EHF and IL-6 were significantly anti-correlated in primary and metastatic prostate cancers. ESE3/EHF bound to the IL-6 promoter and repressed IL-6 transcription (150). Moreover, IL-6, phosphorylated STAT3 and STAT3 transcriptional activity were consistently upregulated in tumor-spheres from ESE3/EHF under-expressing tumor cells, in line with aberrant activation of IL-6/JAK/STAT3 pathway in prostate CSC. To test the effect of antagonizing IL-6/JAK/STAT3 pathway in ESE3/EHF under-expressing tumors, we used the JAK2 inhibitor NVP-BSK805 (150). NVP-BSK805 significantly reduced tumor-sphere formation in ESE3/EHF low expressing models. Moreover, treatment with NVP-BSK805 inhibited growth of tumor xenografts and self-renewal capability of tumor-sphere cells derived from ESE3/EHF knockdown models, indicating that the CSC compartment was compromised persistently by disrupting the IL-6/JAK/STAT3 axis in the context of ESE3/EHF^low^ tumors (150). Many JAK inhibitors are currently in clinical trials for oncological and non-oncological indications (181), making their use for counteracting CSC expansion in specific subtypes of prostate cancer a reasonably testable hypothesis. Collectively, these data indicate that ESE3/EHF activity is essential to maintain the balance between differentiation and self-renewal in the prostate epithelium and that loss of expression of this transcriptional regulator characterize aggressive tumors specifically susceptible to approaches aimed at restoring the tumor suppressor function of ESE3/EHF.

## Conclusions

In recent years, a growing body of evidence has accumulated on the role of CSC in the genesis and progression of prostate cancer. Prostate CSC play a pivotal role in castration-resistance and phenotypic plasticity that underlie treatment failure and disease recurrence in advanced stage patients. Therapies targeting prostate CSCs can lead to effective treatment for these patients. Anti-CSC strategies should complement the current therapeutic approaches that aim at reducing AR-dependent and proliferating bulk tumor cells. The dissection of the molecular mechanisms controlling the dynamic phenotypic changes that characterize the CSC subpopulation is a mandatory prerequisite to design precise therapeutic interventions aimed at eradicating the CSC. Stem cell reprogramming factors, transcriptional regulators, and epigenetic effectors sustain the maintenance and expansion of prostate CSC and may represent valid therapeutic targets. We have shown that blocking expression and function of transcription factors that are aberrantly upregulated in prostate CSC derived from human cell lines, xenografts and GEM models results in substantial depletion of the CSC subpopulation and severe impairment of the self-renewal and tumorigenic capability. These approaches based on the use of small-molecule chemical inhibitors or synthetic siRNA provide innovative strategies to disrupt the pro-tumorigenic signaling sustaining the prostate CSC phenotype. Nevertheless, despite the enormous progress seen in the last decades, many questions on the heterogeneity and plasticity of prostate CSCs and their evolution during tumor progression and treatment remain open and the results will influence the successful implementation of anti-CSC therapies (1, 3, 5, 48). The application of emerging technologies such as single-cell genomics and spatial transcriptomics (182–186) will allow addressing the important questions of stem cell niche composition, anatomical location, biological and genomic heterogeneity of prostate CSCs in longitudinal studies in mouse models and human samples. Genomic and proteomic approaches may lead to the development of specific CSC signatures to apply to preclinical models and human samples and probe the CSC population and characterize their heterogeneity and evolution during the course of the disease and in response to treatments (31, 95, 187). Likely, combinations of standard therapies targeting bulk tumor cells with more selective anti-CSC therapies would be the most reliable treatment approach for most patients. Combined targeting of multiple CSC pathways might also be required to achieve effective control of the CSC subpopulation within highly heterogeneous tumors and avoid CSC escape. Properly designed preclinical studies and clinical trials should investigate the feasibility and efficacy of the diverse strategies matching the genotypic and epigenetic features of the tumors.

## Author Contributions

GC, DA, DS, RV, JM, AK, SM, GMC, and CC reviewed the literature, analyzed the data, and contribute to writing the manuscript.

### Conflict of Interest Statement

The authors declare that the research was conducted in the absence of any commercial or financial relationships that could be construed as a potential conflict of interest.
